# Bedside to bench and back again-translational research in interventional pulmonology

**DOI:** 10.1097/MCP.0000000000001125

**Published:** 2024-10-16

**Authors:** Beenish Iqbal, Hee Jae Choi, Nikolaos I. Kanellakis, Jason Akulian, Najib M. Rahman

**Affiliations:** a Oxford Respiratory Trials Unit, University of Oxford, Oxford, UK; b PGY-5, Division of Pulmonary and Critical Care, University of North Carolina, Chapel Hill, North Carolina, USA; c Chinese Oxford Institute, University of Oxford, Oxford, UK; d Interventional Pulmonology and Pulmonary Oncology, Division of Pulmonary and Critical Care, Lineberger Comprehensive Cancer Center, University of North Carolina, Chapel Hill, North Carolina, USA; e Professor of Respiratory Medicine, Director Oxford Respiratory Trials Unit, Nuffield Department of Medicine, University of Oxford, Oxford, UK

**Keywords:** interventional pulmonology, lung cancer diagnostics, pleural disease, translation

## Abstract

**Purpose of review:**

Translational research in Interventional Pulmonology has made significant advances in recent years, ranging from novel biomarkers and imaging to practice-changing clinical trials in lung cancer and pleural disease. This review article aims to summarize key research studies in the field to understand the latest published evidence and to highlight areas of growing academic interest.

**Recent findings:**

In lung cancer, the role of novel imaging and biomarkers and their potential utility in early lung cancer diagnosis will be highlighted. In pleural disease, less invasive/conservative treatment in pneumothorax, early aggressive treatment in pleural infection along with novel biomarkers, and the shift beyond drainage strategies in malignant pleural effusion and mesothelioma will be discussed.

**Summary:**

This overview of translational research in the field of interventional pulmonology will ultimately help to highlight the gaps in current evidence to promote research in areas of clinical significance.

## INTRODUCTION

The recognition of interventional pulmonology (IP), as a subspecialty of respiratory medicine is a relatively recent phenomenon but the procedures and practices of IP have been around for centuries [1]. Hence, research in IP has not followed a classical ‘bench-to-bedside approach’ and has rather utilized an inverted approach of ‘bedside-to-bench and back’. In the last few decades, the focus of IP research was experiential reports of new technology use and refinement of diagnostic and therapeutic methods. More recently, research efforts have begun to better understand the pathophysiological underpinnings of diseases which are playing a foundational role in guiding newer diagnostic and therapeutic approaches. This article will aim to summarize key recent and current translational research, both early (basic sciences) and late (clinical), in the field of lung cancer diagnostics and pleural disease [2]. 

**Box 1:**
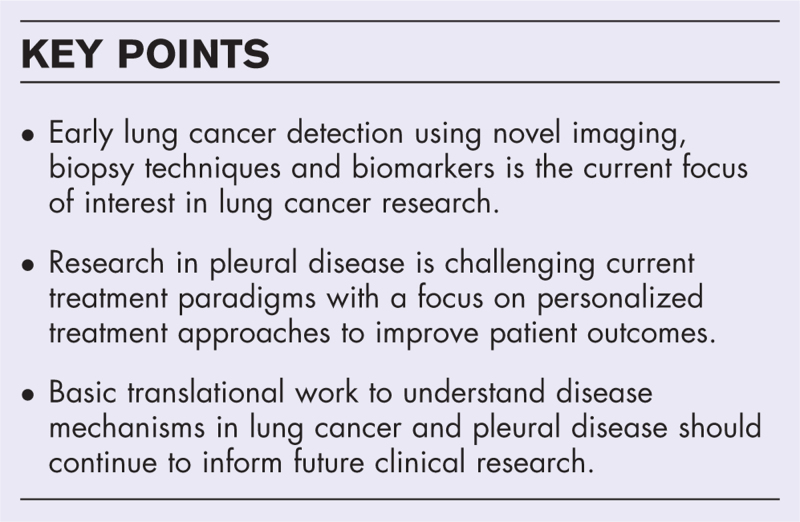
no caption available

## RESEARCH IN LUNG CANCER DIAGNOSTICS

Lung cancer remains the leading cause of cancer death, accounting for 20.4% of all cancer deaths in 2024 [3]. Efforts to screen for, diagnose, and treat lung cancer in its early stages have led to an increase in peripheral pulmonary nodule (PPN) detection [4]. Although low-risk PPNs may be observed, current guidelines recommend that intermediate and high-risk PPNs should undergo biopsy and/or resection to establish a diagnosis of early-stage lung cancer, improving cancer-specific and overall mortality. Given the significant impact on patient survival, research efforts are being expended to enhance the risk stratification and diagnosis of early-stage lung cancer.

### Bronchoscopic advances

Computed tomography-guided transthoracic needle biopsy (CT-TTNB) has long been considered the gold standard for diagnosing PPNs with high rates of published diagnostic yield, albeit with rates of higher procedural complication than bronchoscopy [5,6]. Over the last few years, the field of IP has witnessed technological advancements including robotic-assisted navigation bronchoscopy (RAB) coupled with peri-operative imaging such as cone beam computed tomography (CBCT) or digital tomosynthesis (DT).

The introduction of RAB with integrated imaging promises the ability to navigate smaller airways and stabilize equipment for biopsies. Retrospective studies and meta-analyses of RAB have reported diagnostic yields between 69% and 84.3% [7,8]. A retrospective comparative study of RAB to CT-TTNB reported similar diagnostic yields between the two sampling modalities [9]. Recently, the first multicentre, prospective, randomized, noninferiority VERITAS trial, directly compared CT-TTNB to electromagnetic navigation (EMN) with DT. Preliminary results have reported noninferiority when comparing EMN to CT-TTNB, but EMN was associated with 25% fewer complications when compared to CT-TTNB [10^▪▪^].

Despite these technological advancements, CT-to-body divergence has remained a limitation to improving the diagnostic yield of RAB. Additional imaging modalities, such as CBCT and DT-based imaging devices, have been studied to improve target localization discrepancies by targeting peri-operative tool-in-lesion localization. A retrospective study of DT in PPN biopsy has reported diagnostic yields of 80%, 84% and 78%, respectively, however outside of VERITAS there remains little or no data prospectively evaluating its effect [11–13]. A retrospective study of RAB with CBCT has reported a diagnostic yield ranging from 77% to 91.4% [14,15].

A new RAB system utilizing integrated DT received FDA clearance in March 2023. In the recent single-centre prospective FRONTIER study, 18 patients with PPN underwent biopsy using this new system with a reported diagnostic yield of 89.5% and an apparently acceptable safety profile [16] albeit with low numbers.

The real-time localization of PPNs with these imaging modalities, in addition to RAB platforms, appears to provide some improvement in PPN diagnosis, but the significant expense of these treatments is a potential limitation to their widespread utilization and access. While these early reports are encouraging, robust clinical data of their clinical utility is lacking with future prospective multicentre comparative effectiveness trials needed to fully adjudicate the benefit of these technologies (RAB and CBCT/DT) alone and in combination [17,18].

### Translating the genome, proteome, and imaging – biomarkers for diagnosing lung cancer

Along with the innovation in mechanical technology for bronchoscopic diagnosis, there is increasing research in noninvasive risk stratification of PPNs. Key areas of investigation include blood-based biomarkers, airway genomic classifiers, and exhaled breath condensate of volatile organic compounds (VOC) to assess the risk of lung cancer in patients with PPNs.

Blood-based biomarkers have been most extensively studied for their potential to stratify PPNs into benign and malignant categories. Developed in 2010, the Nodify CDT (BioDesix, Inc. Louisville, CO, USA), is a blood-based test that measures seven autoantibodies associated with tumour antigens and was developed as a ‘rule-in’ test with high specificity (94%), low sensitivity (19%) and high positive predictive value (89%) [19]. Despite this seemingly very good performance profile in ‘ruling-in’ malignancy, the company's efforts pivoted to the development of a ‘rule-out’ test. The Nodify XL2 (BioDesix) test, was subsequently introduced in 2013, as a blood-based proteomic test that measures the abundance of two plasma proteins combined with five clinical risk factors, to determine the likelihood of lung cancer. This test was developed as a ‘rule-out’ test with a high sensitivity (97%), low specificity (44%) and high negative predictive value (98%) [20]. Both tests are offered together but can also be ordered separately. An ongoing prospective, multicentre, randomized controlled trial that will evaluate the clinical utility of the Nodify XL2 proteomic classifier is scheduled to complete enrolment in the next 12–24 months, with the goal to assess the utility of this biomarker as a clinical decision aid in the risk stratification of PPN [21].

For PPNs caused by a benign pathology, particularly those found in regions with endemic mycoses, serum antibody testing has been shown to improve diagnostic accuracy, reduce time to diagnosis, and decrease the need for invasive procedures [22]. Research also has been shifting towards not only identifying blood-based biomarkers but combining them with clinical factors and identifying the optimal machine learning algorithm, to determine risk for malignancy. In a recently published study, a multidisciplinary team reported the development of a support vector machine algorithm incorporating seven plasma proteins and clinical factors, achieving improved risk stratification compared to the Mayo Clinic Model [23].

The use of airway and now nasal epithelium has also been studied with epithelial genomic classifiers being utilized to quantify the airway field cancerization effect [24,25]. The Percepta genomic sequencing classifier (Veracyte, Inc., San Francisco, CA, USA) identifies gene expression alterations in airway epithelial cells amongst patients with a history of tobacco use. This classifier risk stratifies patients with PPN based on the changes in their cellular genome. Previously reported with positive findings in the AEGIS-1 and AEGIS-2 studies [26,27], clinical adoption of genomic classifiers has been slow as real-world outcomes have not consistently matched those previously reported. More recently, a nasal-epithelial cell-based genomic classifier has been introduced with the hope that this approach could serve as an accurate biomarker for the risk stratification of PPN, potentially obviating the need for bronchoscopy in patients stratified to low risk with the cohort study demonstrating 97% sensitivity and 40% specificity [28]. The NIGHTINGALE prospective clinical utility study is currently undergoing enrolment to demonstrate its effects on patient care and outcomes [29].

Finally, the assessment of VOCs found in exhaled breath condensate to risk stratify and/or diagnose lung cancer has also begun to generate significant interest. Based on the idea of canine detection of VOCs, this area of study remains early in its maturation curve [30]. In a recent multicentre validation study of an ‘electronic-nose,’ the study team reported the device as being effective in reliably distinguishing between patients with nonsmall cell lung cancer and healthy individuals [31]. The multicentre study consisted of one training cohort and a validation cohort; the groups had sensitivities and specificities of 93%, 54%, 88% and 48%, respectively. Early results are promising and will hopefully be followed by more robust studies to expand a new field of lung cancer detection, with the added significant attraction of it being an easy and noninvasive test.

## RESEARCH IN PLEURAL DISEASE

Research in pleural disease has been predominantly late translational, i.e. clinical phase III randomized controlled trials (RCTs), which have defined modern management of pleural disease. Below is a summary of key recent research advances in the field.

### Pneumothorax

The research in primary spontaneous pneumothorax (PSP) has seen a paradigm shift in recent years with the focus moving away from drainage of air [32]. A recent RCT by Brown *et al.* demonstrated that conservative care in PSP was noninferior to chest drain insertion in the radiological resolution of pneumothorax and ∼85% of patients required no intervention in the conservative arm [33]. Although conservative care has been around since the 1960s [34], there is a renewed interest in this area and a current trial, CONSEPT, is investigating the role of conservative care in patients with *symptomatic* large PSP using a patient-focused primary outcome i.e. the need for further interventions in the first 30 days. Similarly, the CoMiTED trial is assessing the efficacy of conservative care in traumatic pneumothorax for the first time, an area which has historically been treated with invasive interventions [35]. If the evidence favours conservative care in pneumothorax, this will lead to a practice-changing shift in current clinical care.

For patients who require chest drainage for pneumothorax, there is a focus on less invasive treatments requiring fewer days in the hospital. The EXPRED trial, the largest to date in the PSP population, has shown that although needle aspiration is noninferior to chest drain in the management of PSP, it results in fewer adverse events and a lower recurrence rate of 20% compared to 27% with chest drain [36]. Hallifax *et al.* have shown in the RAMPP trial that ambulatory management of PSP using a Heimlich valve device can significantly reduce the duration of hospitalization compared to chest drain insertion in the first 30 days postrandomization [0 vs. 4 days, median diff 2 days, 95% confidence interval (CI) 1–3, *P* < 0.0001] [37]. In Secondary Spontaneous pneumothorax, the PRINCE-SSP trial is currently investigating the role of therapeutic aspiration compared to chest drain in reducing the hospital length of stay.

Newer pilot studies in pneumothorax are underway to investigate the mechanism of breathlessness [38] and novel noninvasive imaging techniques to detect air leak. Thus, the future holds promise for exciting insights into the management of pneumothorax.

### Pleural infection

Pleural infection is a serious illness with high morbidity and mortality that has remained unchanged for decades [39]. The standard treatment has been chest drainage and antibiotics with surgery reserved for complex cases [40]. Since the landmark MIST-2 trial, the use of intrapleural enzyme therapy or IET (combination of tPA/DNAse) in the treatment of complicated pleural infections is the most important translational leap in this field [41]. IET acts as an adjunct to the standard treatment, has a favourable safety profile [42] and around 90% of complex cases get treated with the IET without requiring surgery [43]. However, IET and surgery are usually reserved for complex cases of pleural infection and there has been an increasing interest to explore if the early introduction of these treatments could improve patient outcomes in pleural infection.

The MIST-3 trial has recently shown the feasibility of comparing the early IET and surgery in the management of pleural infection with some early signals towards less pain and quick recovery with IET [39]. Based on this, the comparison of early IET and surgery will be conclusively studied in MIST-4, a definitive RCT aiming to recruit >600 patients. The FIVERVATS trial in Denmark is answering a similar question in the Danish population [44]. The result of these trials will be revolutionary to improve outcome of patients with pleural infection.

Pleural infection in the context of indwelling pleural catheters (IPCs) is a poorly understood area [45]. A current AMPLE-4 trial is recruiting patients for prophylactic topical antibiotics with IPC to investigate infection prevention [46]. If proven beneficial, this will be a new era of preventive treatments for IPC-related infections with significant clinical utility.

In risk prediction for pleural infection outcomes, the RAPID score is the only prospectively validated score to predict mortality in pleural infection [47]. The role of RAPID-driven management of pleural infection will be studied in the upcoming RAPTOR-f study, which will assess the feasibility of randomizing patients to a RAPID score driven treatment pathway vs. usual care.

Early translational work in pleural infection exploring the role of biomarkers and newer microbiological culture techniques is worth mentioning. PAI-1 levels in pleural fluid demonstrated a significant association with increased length of hospital stay and 1-year mortality in pleural infection [48]. Similarly, suPAR levels can provide an early prediction of complications in pleural infection [49]. The TORPIDS study investigated the promising role of 16S rRNA sequencing to improve the microbiological yield in pleural infection [50]. However, these biomarkers and assays require robust investigation for clinical utility in prospective studies before being translated into clinical practice.

### Malignant pleural effusions and mesothelioma

The research in the management of malignant pleural effusion (MPE) has mainly focused on devising the ideal drainage strategy to improve breathlessness in patients with advanced cancer and poor life expectancy [51]. Chest drain with talc pleurodesis (TP) and indwelling pleural catheter (IPC) are used for the ‘definitive’ management of recurrent MPE. Multiple RCTs have demonstrated that TP and IPC provide similar symptomatic benefits in MPE with no superiority of either treatment [52–55]. The OPTIMUM trial showed that there is no difference in the quality of life of patients treated with TP or IPC [56]. Two recent trials, TACTIC and AMPLE-3 have compared the use of thoracoscopic talc poudrage + IPC with standard care and poudrage via VATS with IPC ± talc, respectively [57,58]. The results of these trials will inform newer strategies for recurrence prevention in MPE.

Despite extensive research in MPE management, the current approach to diagnosis and treatment of MPE is one-size-fits-all, with a need for a more ‘personalized’ approach. In this context, the REPEAT study is developing a prediction score for patients with fast or slow MPE recurrence rates to guide treatment. The prognostication in MPE using predictive scores like LENT, PROMISE and BLESS is an important area of translational research that can guide patient-centred treatment decisions in MPE [51].

In reducing the time to diagnosis and treatment, the STREAMLINE study is investigating the feasibility of undertaking a large RCT of combined pleural biopsy and IPC as an index procedure compared to standard pleural aspiration in MPE. Similarly, the diagnostic yield of cell-free DNA for molecular markers in MPE is comparable to pleural biopsy in case series with the potential of further reducing the time to diagnosis in places with limited access to pleural biopsy [59]. Together, these approaches have a huge potential to change practice in MPE management.

In the field of mesothelioma research, PREDICT-Meso is an international programme aiming to identify premalignant drivers and develop faithful pleural mesothelioma models and analysis pipelines. The MARS-2 trial has conclusively shown that surgery is harmful in mesothelioma with worse prognosis and more serious adverse events compared to medical management [60]. Thus, the need for newer dedicated medical treatments has led to a renewed interest in intrapleural drug treatments owing to the localized nature of mesothelioma [61]. The currently recruiting MITOPE study, the first-in-human trial in patients with progressive MPE and mesothelioma, is investigating the anticancer effects of intrapleural administration of a novel oncological drug RSO-021. This is the beginning of an era of targeted anticancer therapies to avoid systemic toxicities.

## CONCLUSION

In summary, translational research in IP has made great strides in recent times with a tremendous impact on patient care, and there is potentially more practice-changing research on the horizon. In lung cancer, the role of novel diagnostic tools requires validation in prospective RCTs to confirm utility and impact on patient outcomes. Research in pleural disease has started to see a shift towards newer diagnostic and treatment approaches driven by an increasing understanding of the underlying disease pathogenesis.

## Acknowledgements

*None.*

### Financial support and sponsorship

*None.*

### Conflicts of interest

*There are no conflicts of interest.*

## REFERENCES AND RECOMMENDED READING

Papers of particular interest, published within the annual period of review, have been highlighted as:

▪ of special interest
▪▪ of outstanding interest
